# Olfactory immune response to SARS-CoV-2

**DOI:** 10.1038/s41423-023-01119-5

**Published:** 2023-12-25

**Authors:** Sebastian A. Wellford, E. Ashley Moseman

**Affiliations:** Department of Integrative Immunobiology, Duke University School of Medicine, Durham, NC USA

**Keywords:** Mucosal immunology, Neuroimmunology, Infectious Disease, Sars-CoV-2, Mucosal immunology, Infectious diseases

## Abstract

Numerous pathogens can infect the olfactory tract, yet the pandemic caused by SARS-CoV-2 has strongly emphasized the importance of the olfactory mucosa as an immune barrier. Situated in the nasal passages, the olfactory mucosa is directly exposed to the environment to sense airborne odorants; however, this also means it can serve as a direct route of entry from the outside world into the brain. As a result, olfactotropic infections can have serious consequences, including dysfunction of the olfactory system, CNS invasion, dissemination to the lower respiratory tract, and transmission between individuals. Recent research has shown that a distinctive immune response is needed to protect this neuronal and mucosal tissue. A better understanding of innate, adaptive, and structural immune barriers in the olfactory mucosa is needed to develop effective therapeutics and vaccines against olfactotropic microbes such as SARS-CoV-2. Here, we summarize the ramifications of SARS-CoV-2 infection of the olfactory mucosa, review the subsequent immune response, and discuss important areas of future research for olfactory immunity to infectious disease.

## Introduction

Remarking upon the intoxicating power that odors hold over our memories, Hellen Keller once said “smell is a potent wizard that transports you across thousands of miles and all the years you have lived”. Unfortunately, the pathway for smell may also transport neuroinvasive pathogens directly into our brains. Nature has exquisitely designed the olfactory system to achieve the sense of smell through a unique and complex anatomical arrangement. The olfactory mucosa (OM), consisting of a pseudostratified epithelium and lamina propria, lines the upper portion of the nasal cavity in the upper respiratory tract (URT). Neurobiologists have studied the biology of olfaction, including the life-long neurogenesis and unique patterning that characterize the olfactory system, for decades. However, very little effort has been made to elucidate how this sensory tissue is protected from pathogens. The pandemic caused by SARS-CoV-2 led to millions of short- and long-term smell loss cases. Indeed, for many people, loss of smell was the most striking first symptom of infection. The olfactory neuroepithelium is home to several unique cell types, but most importantly, it contains numerous olfactory sensory neurons (OSNs). The cell bodies of these neurons reside within the neuroepithelium, where they extend their dendrites with extensive cilia directly into the mucosal airway. If these cilia encounter an odorant, the OSN can rapidly relay a signal along the length of its axon. OSN axons converge to form bundles that then traverse holes in the cribriform plate of the skull, terminating directly in the olfactory bulb of the brain [1]. As a result, olfaction is a sensitive and efficient process for communicating information about the external environment. However, precisely because the OM simultaneously straddles the airway and the brain, microbes can subvert this biology to directly invade the CNS, bypassing conventional brain barriers with disastrous consequences, including fatal meningitis and encephalitis. Even for nonneuroinvasive pathogens such as SARS-CoV-2, local olfactory infection can drive inflammatory reactions that impact the adjacent CNS.

The olfactory mucosa can therefore be considered a mucosal barrier for the brain. Moreover, it is an important component of the upper airway. The URT is frequently thought to be a relatively homogeneous tissue, but unlike the respiratory lining of the URT, the OM is a distinct neuronal tissue with vastly different cell types and immune considerations. The OM plays an important role in the pathogenesis of respiratory diseases, even if CNS symptoms do not occur. The nose is the entry site for most respiratory pathogens, many of which have known olfactotropism; others have undefined olfactotropism. The immune response in the OM can therefore be critically important for preventing pathogen dissemination to other tissues in the body. Because OM pathogen replication may also serve as a pathogen reservoir, the OM immune response likely also serves to reduce transmission between individuals. In addition, infection-related OM damage impairs the ability to smell, an outcome that negatively impacts quality of life and the ability to sense environmental danger. Immunity against olfactotropic infections is therefore essential to prevent CNS neuroinvasion and respiratory pathogen dissemination and transmission and to protect the sense of smell itself.

Anosmia, smell loss without acute airflow blockage, was quickly recognized as a negative effect of COVID-19 but also indicated that the virus distinctly impacted the olfactory mucosa. The ubiquity of olfactory infections during the COVID-19 pandemic has stimulated interest in the OM as an immunological tissue. Unfortunately, this interest has served to highlight how poor our understanding of immune responses in the olfactory system is. As an additional side effect of the pandemic, researchers and the public are beginning to pay more attention to other potentially olfactotropic pathogens. Indeed, many respiratory and neurotropic pathogens are able to infect the OM, but the attention that the pandemic brought to olfactory infection has focused an important spotlight on this understudied URT region. Nevertheless, due to difficulty in obtaining olfactory biopsies and how infrequently the olfactory system is considered clinically, many common infections may impact the OM in ways we do not yet understand. Furthermore, many animal models of infection fail to establish olfactotropism in airway diseases. We currently are at an inflection point in the study of olfactory disease pathogenesis, as recent studies in animals and in humans have begun to emphasize the importance of the OM immune response.

Olfactory immunology has implications for several topics of clinical importance, including vaccination, encephalitis and meningitis, postviral olfactory dysfunction, microbial transmission, innate and adaptive immunity, and neuroimmunology. This review summarizes infectious diseases and subsequent immune responses in the olfactory mucosa. We directly address SARS-CoV-2 and similar viral pathogens that infect the olfactory mucosa. Olfactory immunity against these microbes is also discussed, and future directions for the nascent field of olfactory immunology are considered.

## Olfactory SARS-CoV-2 infection

### Human pathogenesis

**Fig. 1:**
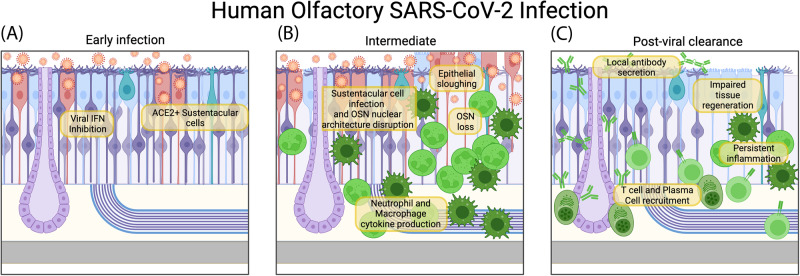
Olfactory immune response to SARS-CoV-2 in Humans. The stages of the immune response to SARS-CoV-2 in the olfactory mucosa. **A** SARS-CoV-2 uses ACE2 to enter sustentacular cells and antagonize the induction of interferons. **B** Infiltrating neutrophils and macrophages produce inflammatory cytokines. Sustentacular cells are lost, epithelial structure deteriorates, and olfactory sensory neurons undergo disruption of nuclear architecture and cell death. **C** After SARS-CoV-2 has been cleared by the immune response, T cells and plasma cells populate the tissue. Plasma cells produce locally protective mucosal antibody. T cells may contribute to sustained inflammation, preventing proper epithelial regeneration in some cases and preventing restoration of the sense of smell

At the onset of the COVID-19 pandemic, as cases spread across the globe, there was fear that the pathogen, similar to other coronaviruses, may have neurotropic tendencies. While pulmonary infection can drive severe disease and death, concern for neurotropism was sparked after olfactory dysfunction emerged as a very commonly reported symptom of COVID-19 [2–4], combined with widespread reports of neurological consequences such as “brain fog”. As a result, several groups have sought to ascertain whether SARS-CoV-2 can invade the CNS through the olfactory nerve. Initially, postmortem samples of human brain and olfactory tissue provided concerning but uncertain evidence regarding the occurrence and relevance of SARS-CoV-2 olfactory neuroinvasion, with the virus occasionally being present in the brain [5–7] of individuals who had died of COVID-19. As expression of ACE2 and TMPRSS2, the host proteins needed for SARS-CoV-2 cell entry, was largely uncharacterized in the olfactory system, it was difficult to determine whether direct OSN infection was possible. Furthermore, Nrp1 was suggested as an alternative entry receptor that may facilitate olfactory infection [8]. Subsequent studies indicated that while these entry proteins are present in the OM, they are primarily expressed on sustentacular cells [8–14]. Of note, *ACE2* was found to be expressed more highly in the URT than in the lungs [15]. Infection or inflammation can upregulate *ACE2* in the epithelium [10], and induced *ACE2* is expressed as the isoform dACE2, which is incapable of binding the SARS-CoV-2 spike protein [16]. Additional analyses of olfactory biopsies have painted a clearer picture of SARS-CoV-2 infection in the olfactory system (Fig. 1). In humans, the virus primarily infects the sustentacular cells of the olfactory mucosa [17–21], which fits with the observed distribution of the ACE2 entry receptor. These cells are the primary structural cell type in the olfactory epithelium, providing support for OSNs and their dendritic projections to detect odors at the air interface. SARS-CoV-2 sustentacular cell infection leads to massive inflammation leading to sustentacular cell death, loss of epithelial tissue structure, and subsequent disruption of OSN nuclear architecture and function [17, 21]. Consequently, without structural support, OSNs can be lost, and the sense of smell is either diminished or completely ablated. Fortunately, direct SARS-CoV-2 olfactory neuron infection rarely occurs, and subsequent neuroinvasion seems unlikely. A critical analysis of studies claiming olfactory neuroinvasion was conducted by Butowt et al. [20]. In relatively rare cases in which SARS-CoV-2 infection of the CNS is identified [6, 7], olfactory neuroinvasion should be considered as one possible route of entry, along with spread across the inflamed blood‒brain barrier and infection of other neuronal cells in epithelial tissues [20, 22–24].

### Animal pathogenesis

**Fig. 2:**
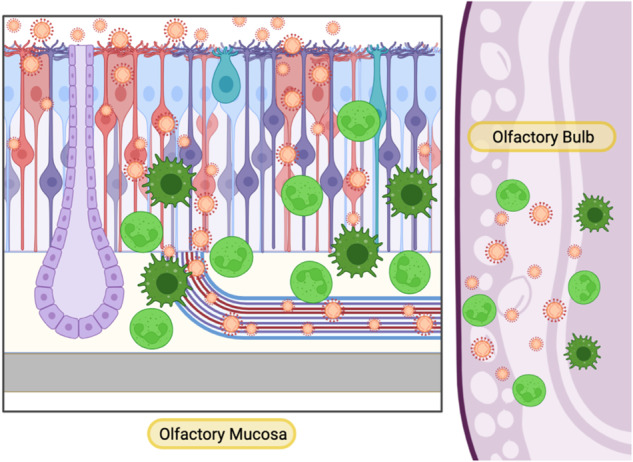
Olfactory neuroinvasion in mammalian SARS-CoV-2. In some animal models, SARS-CoV-2 is able to invade the brain through the olfactory nerve. The virus infects sustentacular cells and olfactory sensory neurons in the mucosa, then travels through olfactory axons, reaching the olfactory bulb of the brain. Immune cells migrate to both the mucosa and the brain, producing inflammatory mediators that can both fight the virus and contribute to destructive neuroinflammation

Overall, SARS-CoV-2 olfactory pathogenesis varies across animal models. Mammalian studies have helped to shed light on SARS-CoV-2 human pathogenesis while also illustrating the dangers of olfactory neuroinvasion. In some organisms, SARS-CoV-2 invades the brain via the olfactory nerve (Fig. 2), raising the concern that future variants may have more neurovirulent tendencies. Indeed, experiments across mammalian models indicate that olfactotropism differs depending on the variant of SARS-CoV-2 [24–26].

Because parental SARS-CoV-2 cannot use murine ACE2 for cell entry, to study SARS-CoV-2 in mice, animals must be engineered to express human ACE2, or a murine-adapted virus must be used. The commonly used K18-hACE2 transgenic mouse was initially generated as a model for SARS-CoV-1 [27] and uses the human keratin-18 promoter to artificially express hACE2 in nearly every epithelial cell, including OSNs and sustentacular cells. As a result, direct OSN infection and consequent CNS pathology are observed following intranasal inoculation [28, 29]. Multiple SARS-CoV-2 variants often result in lethal disease [30]. In addition to olfactory transmucosal neuroinvasion, these mice lose their sense of smell. This smell loss persists for several weeks following infection and likely reflects widespread damage to the olfactory epithelium caused by sustentacular and OSN infection [31]. K18-hACE2 mice experience extensive olfactory tissue damage characterized by the “sloughing off” of the epithelial layer, consistent with other observations of aggressive olfactory infections [28, 32]. Interestingly, aerosolization of SARS-CoV-2 as opposed to intranasal droplet instillation changes its olfactory pathology in K18-hACE2 mice [33]. Aerosolized inoculation results in lower viral titers in the nasal turbinates, no viral spread to the olfactory bulb, and greater viral replication in the lungs and respiratory tract, more closely mirroring human infection. These mice still develop smell impairment, suggesting that nasal infection in the absence of CNS neuroinvasion can still drive olfactory damage and functional dysosmia [33]. The reasons why aerosolization does not result in neuroinvasion remain unclear but will be critical to understanding factors that may heighten neuroinvasive tendencies in other viruses.

Another mouse model in which hACE2 expression is driven by the endogenous mouse *Ace2* promoter also shows infection of the olfactory epithelium and fatal neuroinvasion [34]. In this model, SARS-CoV-2 accesses the brain by infecting olfactory neurons, resulting in lethal cachexia, hypoxemia, and respiratory failure independent of lung infection. Selective expression of hACE2 in the olfactory epithelium and in neurons is sufficient for these phenotypes, illustrating that SARS-CoV-2 can cause severe illness even without lower respiratory tract infection [34]. Other murine models have been used to model different aspects of COVID-19, including mouse-adapted SARS-CoV-2 strains [31] and mice that express hACE2 via AAV-controlled expression [6]. Chimeric viruses, such as VSV-SARS-CoV-2 S, which expresses the SARS-CoV-2 spike protein on a vesicular stomatitis virus (VSV) backbone, have been useful for studies of the humoral immune response to SARS-CoV-2 in mice [35, 36].

A more “natural” model of SARS-CoV-2 olfactory neuroinvasion occurs in Syrian golden hamsters (Fig. 2). Similar to humans, SARS-CoV-2 was initially observed to infect sustentacular cells in the olfactory epithelium, causing massive immune infiltration and desquamation [37, 38]. However, olfactory neurotropism of SARS-CoV-2 in hamsters seems to be highly dependent on the viral isolate, as some have been shown to replicate in OSNs without CNS infection [39, 40], while others infect OSNs and invade the CNS [41] Some subsequent variants, such as Delta in one study [25] and D614G in another [42], cause even more OSN infection and CNS neuroinvasion, with variants such as Omicron having fewer of these tendencies [25, 42]. More recent data detected SARS-CoV-2 in the olfactory bulb for all five variants tested, though infection led to smell loss in only some variants [43]. Consistent with mouse data [33], this suggests that viral-induced anosmia may be independent of neuroinvasive capacity [43]. Indeed, hamsters frequently have lasting olfactory perturbations following SARS-CoV-2 infection, indicating that they may be useful models for post-COVID olfactory dysfunction [44, 45].

Nonhuman primates, especially macaques, have been used to test the immune response to SARS-CoV-2 and vaccine candidates, but direct examination of the olfactory mucosa has been infrequently conducted in these animals. SARS-CoV-2 infects the nasal passages of macaques [46–48] but does not seem to be neuroinvasive, though some studies have detected low levels of SARS-CoV-2 in olfactory CNS regions [49, 50]. SARS-CoV-2 infects many wildlife species and domesticated animal populations [51], but little is known about olfactory pathogenesis in other mammalian hosts. However, given the high fatality rate in wildlife populations such as deer and mink [52, 53], the frequency at which olfactory neuroinvasion occurs in these species should be investigated.

These animal data suggest that future variants of SARS-CoV-2 (or, more generally, future pandemic coronaviruses) may have increased olfactory neurotropism. How can we prepare for an emergent respiratory pathogen that may also cause catastrophic CNS infection? We must design vaccines that capably protect the OM. Critically, these immunizations should focus on generating mucosal antibodies since OSNs are directly exposed to the airway. We should also investigate therapeutics that target nasal viral replication shortly after exposure, as well as drugs that may effectively treat viral meningitis and encephalitis. Furthermore, SARS-CoV-2 has long-term neurological and olfactory consequences, for which there is little clinical recourse. A better understanding of olfactory immunity in general is needed to address all these challenges. To these ends, what lessons can we take from the COVID-19 pandemic? Below, we summarize what is known about the immune response to SARS-CoV-2 in the olfactory system.

### Innate immune response

SARS-CoV-2 gains a foothold in the host due to its ability to limit the early innate immune response [54] (Fig. 1a). By dampening the interferon (IFN) response in nasal epithelial cells [55, 56], SARS-CoV-2 is able to replicate quickly, often becoming infectious before any symptoms are apparent. However, if innate immunity is activated quickly, it can significantly limit viral dissemination. Furthermore, one study in macaques showed that mild SARS-CoV-2 can be cleared from nasal tissue prior to the arrival of T cells, suggesting that once activated, the innate response can be quite effective [57]. Studies of human nasal samples indicate that a poised immune state, with higher basal levels of IFN and PRR expression, may protect children from severe COVID-19 [58, 59]. Intranasal administration of IFN-based therapeutics, either prophylactically or shortly after infection, has been shown to limit SARS-CoV-2 replication in nasal passages, but differences between respiratory and olfactory IFN responses and viral replication have not been directly measured [60, 61], making it difficult to interpret whether there may be tissue-specific effects of IFN in the URT. Once viral-induced inflammation begins to occur, circulating immune cells infiltrate the infected OM to combat the virus (Fig. 1b). These cells are predominantly neutrophils and macrophages, and their arrival into the tissue is accompanied by an increase in cytokine production [21, 31, 37, 38, 62]. These cytokines likely play important roles in both antiviral effector mechanisms and wound healing, but some evidence indicates that neutrophilic inflammation can actually exacerbate tissue damage and increase OM viral replication [38]. Nasopharyngeal swabs of humans primarily contain cells of respiratory origin while neglecting olfactory inflammation, but related studies have provided evidence to suggest that activated macrophages and neutrophils are involved in COVID-19 nasal inflammation and correlate with disease outcomes [56, 63–65]. Overall, neutrophils and macrophages seem to be important players in the olfactory COVID innate response, but whether they are productive or deleterious likely depends on the magnitude of the response and the specific cytokines expressed by these cells.

### Adaptive immune response

An effective adaptive immune response in the olfactory mucosa is essential to clear SARS-CoV-2 and protect against future reinfection [66–69]. However, as the COVID-19 pandemic continued, it became apparent that prior infection or immunization provided significant protection from severe disease but did not prevent reinfection [70–73]. This can be partially explained by several factors, including limited neutralization capacity of anti-Spike antibodies, emergence of new variants, waning of the initial immune response, and incomplete herd immunity [74, 75]. However, breakthrough infections commonly present with upper respiratory symptoms and fewer symptoms in the lower respiratory tract [70]. Could immune protection in the nasal passages be incomplete, and if so, is this a phenomenon specific to the olfactory mucosa?

Several SARS-CoV-2 studies have suggested that blood-borne antibodies are incapable of protecting the upper respiratory tract from infection. Passive antibody transfer, while preventing lung SARS-CoV-2 infection, fails to protect the nasal passages, as virus can still be detected within nasal washes and within the nasal turbinates, including olfactory regions [36, 76]. Accordingly, many vaccines induce strong humoral protection of the lungs while insufficiently protecting nasal viral replication across animal models [36, 46, 76–83].

Our recent work has demonstrated why serum antibodies cannot extend protection to the entirety of the nasal airway [84]. We showed that circulating antibodies readily access and protect the respiratory mucosa within the nasal passages but are excluded from entering the olfactory tissue. This is due to the presence of the blood-olfactory barrier (BOB), which forms a tight endothelial barrier to segregate the olfactory mucosa from circulation [84]. As a result, even with high titers of blood-borne antibodies, the olfactory portions of the URT can be left exposed to infection.

Nonetheless, despite the presence of the BOB, the OM can still be protected from rechallenge. Our data and that of others have further shown that plasma cells can home to the OM, residing within the tissue to directly secrete antibodies to the mucosal surface, bypassing the BOB [80, 84] (Fig. 1c). Importantly, not all immunization approaches generate these olfactory plasma cells. Many conventional adjuvants, such as alum and TLR ligands, are incapable of driving plasma cell homing [84].

These data are consistent with studies that demonstrate protection of the upper respiratory tract from SARS-CoV-2 infection (Fig. 1c). Immunization strategies such as nonconventional adjuvants [77, 84], alternative antigen vectors [79, 80, 85–87], or intranasal administration [78, 88–90] have all shown promise in providing superior immune protection of the nasal passages, though careful differential analysis of olfactory and respiratory tissues has not been performed. The exact signals that dictate protective OM plasma cell homing and protection remain to be precisely identified, but the discovery of the BOB has important implications for the design of vaccines that aim to protect the olfactory mucosa. In addition, the relationship between tissue plasma cells and those in other mucosal tissues or bone marrow should be studied to identify how humoral immunity may be unique in the OM or reveal broadly applicable lessons.

Mucosal protection in the nasal turbinates is thought to be linked with the ability of vaccines to elicit antiviral IgA. Accordingly, mucosal IgA production is associated with SARS-CoV-2 protection following mRNA vaccination and prior SARS-CoV-2 infection [91–96]. Eliciting mucosal IgA is one reason intranasal vaccination approaches gained intense interest during the COVID-19 pandemic [97, 98], though parenteral immunizations have also been shown to induce mucosal antibodies in some cases [77, 91, 92]. While there are clearly important roles for secretory IgA in protection against mucosal pathogens, our recent work has shown that IgA production is not needed to protect the olfactory mucosa against viral rechallenge [84]. These data suggest that rather than a specific antibody isotype, the most important correlate for protecting the olfactory mucosa is local antibody production. This is in agreement with recent data that IgG antibodies can be detected in nasal secretions and may play a role in protection against SARS-CoV-2 [65, 83, 95, 99, 100].

Protection of the OM is not dependent on humoral immunity alone; T cells also play an important role in defense against COVID-19. T cells home to the OM following SARS-CoV-2 infection and remain in the tissue after viral clearance [101–103] (Fig. 1c). Similarly, after vaccination, antigen-specific CD8^+^ and CD4^+^ T cells can migrate to the nasal passages and reside long term [87, 103, 104], limiting viral replication upon SARS-CoV-2 challenge [105]. These T cells have a repertoire that differs from that of circulating T cells, suggesting an independent and functionally distinct nasal T-cell response [103]. Similar to the olfactory plasma cell response, T cells seem to differentially home to the nasal mucosa in response to infection or vaccination. Determining the signals that best elicit olfactory T cells is of great importance, especially for protection against future SARS-CoV-2 variants or other viruses that may have greater T-cell epitope conservation than B-cell epitope overlap [106]. While nasal, and especially olfactory, T cells have not been frequently analyzed, some studies in animals and humans offer clues for what immunizations may best stimulate these cells. In humans, infection generates the largest numbers of nasal T cells [101], particularly in combination with prior vaccination [103]. Parenteral immunization with mRNA vaccines, on the other hand, recruits very few T cells to the nasal mucosa [104], raising doubt as to whether parenteral vaccines can generate functional T-cell-mediated protection of the olfactory tissue [107]. As persistent antigen and inflammation in mucosal sites is known to greatly enhance T-cell recruitment and resident memory formation [108], it is logical that infection and local nasal immunization will be most effective at forming a robust nasal T-cell compartment. Consistent with this, viral-vectored intranasal vaccination approaches in mice have been shown to induce SARS-CoV-2-specific nasal T cells [87], and a intranasal vaccination approach led to a similar result in macaques [109]. However, as mentioned above for B cells, adjuvant signals and lymph node interactions can influence the ability of T cells to home to barrier sites [110]. It is possible that infection, unlike immunization, is also better able to induce signals that prime T cells for mucosal homing. Decoding these signals may inform adjuvant approaches that could improve the ability of parenteral vaccinations to drive T-cell homing to the olfactory tissue. Last, while olfactory T cells are likely important for viral clearance and protection during future infection, they may also have undesirable impacts. Indeed, evidence suggests that T-cell-driven inflammation can impair olfactory recovery, emphasizing that the T-cell response in the olfactory mucosa must be tightly regulated [102].

### Consequences of olfactory infection

In many cases, COVID-19 has long-term effects following the acute disease stages. In the olfactory system, this most clearly manifests as intermediate or long-term smell loss [111–113], even following mild infection [114]. Clinical data show that while many patients quickly recover their ability to smell, others do not [2, 3], even up to two years after infection and in the apparent absence of infectious virus [115–117]. What could explain the differences in this regenerative capacity? Evidence suggests that sustained inflammation may prevent olfactory stem cells from repopulating the tissue with functional neurons [118] (Fig. 1c). While initial OM destruction is mediated by sustentacular cell death and infiltrating neutrophils [38], long-term dysosmia coincides with persistent inflammation from T cells and NK cells that express IFN-γ accompanied by changes in local myeloid populations. These immune cells appear to signal to sustentacular cells and olfactory stem cells, shifting them away from a regenerative state [102]. Host genetics also may impact the propensity for smell loss [119], and a variety of treatment options are being attempted clinically, from steroids to olfactory training to platelet-rich plasma [120–122]. However, given the role of sustained inflammation in dysosmia, immunomodulatory approaches may also be considered.

Moreover, sustained inflammation in the olfactory mucosa can alter the state of the CNS, even if the virus itself never reaches the brain parenchyma. In postmortem biopsies, COVID-19 patients showed elevated IFN and inflammatory gene expression in the olfactory bulb [44]. Consistent with this, patients with olfactory dysfunction have detectably larger olfactory bulbs measured by MRI [123, 124]. This prolonged inflammation is likely driven by local cytokines and resident immune cells, but it remains possible that as a neuronal tissue, the olfactory mucosa garners a degree of “immunoprivilege”, allowing it to serve as a long-term viral reservoir. Supporting this hypothesis, nasal swabs have identified SARS-CoV-2 RNA in “long-term viral shedders” several months after the initial infection has subsided [125, 126], and viral antigen has been reported in the OM of some, but not all, patients long term [41]. While replicating virus has not been detected in these patients, it is feasible that low levels of virus may be persistently harbored in the olfactory mucosa beyond the reach of typical nasal swabs. Incomplete viral clearance might explain why inflammation is often sustained in patients with olfactory dysfunction. As mentioned above, more recent variants of SARS-CoV-2 are differentially neurotropic and olfactotropic. Measuring smell loss, either through self-report surveys or through clinical testing, is often difficult on an individual basis, but population-level monitoring of anosmia may be useful for predicting future waves of SARS-CoV-2 and other pandemic viruses [127].

SARS-CoV-2 infection of the olfactory mucosa may contribute to other symptoms of long COVID. Patients report cognitive impairment, headache, brain fog, memory impairment, and anosmia as major symptoms [128–130]. These symptoms are now broadly included under the term “postacute sequelae of SARS-CoV-2” (PASC) [131], but early and continued reference to “NeuroCovid” highlights a core set of neurological consequences associated with SARS-CoV-2 infection [132, 133]. Animal models in which SARS-CoV-2 demonstrates olfactory neuroinvasion have shown inflammation of the olfactory bulb and other brain regions [42, 44] (Fig. 2). SARS-CoV-2 neuroinvasion can also induce gene signatures and pathologies associated with neurodegenerative disorders such as Alzheimer’s disease [134]. Neurological manifestations have been implicated in even mild cases of COVID-19 [135], suggesting that the absence of direct neuroinvasion does not prevent peripheral infection from impacting the CNS. While vascular inflammation [136] likely contributes to these symptoms, inflammation of the airway [137, 138], including the olfactory tissue, may contribute to neurologic disturbances. Accordingly, some studies have demonstrated olfactory bulb inflammation without direct neuroinvasion by SARS-CoV-2 [31]. It is important to note that anosmia itself might contribute to cognitive dysfunction, as the sense of smell contributes to neurological and psychological well-being [139].

## Other olfactotropic viral infections

While SARS-CoV-2 has become the most high-profile pathogen with olfactory implications, many viral pathogens with pandemic potential have shown olfactotropic tendencies with and without clear CNS neurovirulence. In this section, we review known major olfactotropic pathogen threats and consider relevant aspects of the olfactory mucosal immune response to each.

### Other SARS coronaviruses

Other coronaviruses have been shown to infect the olfactory mucosa, including SARS-CoV-1 [140], which does not penetrate the CNS in WT mice but is capable of olfactory neuroinvasion in the K18-hACE2 model [140]. An autopsy study of SARS patients detected the virus in the CNS, but the nasal mucosa was not sampled; thus, the method of neuroinvasion was unknown [141]. Human coronavirus (HCoV)-O43 is frequently detected in brain autopsies of deceased patients [142], and intranasal installation of HCoV-O43 in WT mice leads to infection of the CNS, seemingly spreading from the olfactory system [143, 144]. The deadly Middle East respiratory syndrome (MERS)-CoV outbreak was associated with numerous neurologic symptoms [145, 146], and a transgenic mouse model of MERS indicated the possibility of olfactory involvement in brain infection [147]. In addition, the betacoronavirus porcine hemagglutinating encephalomyelitis virus (PHEV) was recently found to invade the CNS via the olfactory and trigeminal nerves during intranasal murine infection [148]. PHEV infection caused inflammatory cell infiltration in the OM and upregulated expression of several inflammatory genes, such as *Cxcl10* and *Ccl5* [148]. Hepatitis viruses are also included in the family *Coronaviridae*, and two murine adapted strains, A59 and JHM, have been shown to invade the brain through the olfactory mucosa following intranasal challenge [149–151]. JHM causes extensive damage to the olfactory mucosa and OSN loss [152], making it a potentially useful model for postviral olfactory dysfunction. Given the frequency of coronavirus epidemic outbreaks in this century, more research on their potential for olfactory neuroinvasion should be conducted. COVID-19 has caused tremendous loss of human life and wellbeing, yet we are still not prepared to develop vaccines or therapies against future olfactotropic and neuroinvasive pandemic coronaviruses.

### Influenza virus

Influenza viruses show variable olfactotropism but are likely the greatest contributor to postviral olfactory dysfunction. Supporting this, analysis of influenza patients shows an inverse relationship between vaccination rates and subjective olfactory dysfunction [153]. Olfactotropism seems to be dependent on the strain, even within the same family. In mice, influenza B/Malaysia/2506/2004 was found to infect OSNs, but these neurons were able to induce an antiviral gene expression program to nonlytically clear the virus before it could reach the CNS [154]. The recombinant R404BP derivative of influenza A/WSN/33 (H1N1) also infects OSNs, but apoptosis is induced in infected neurons to prevent spread to the brain [155]. Influenza A/Puerto Rico/8/34 (H1N1), also known as PR8, is the most widely used murine influenza model, and while some studies have reported PR8 antigen in the olfactory bulb of infected mice [156, 157], PR8 does not productively infect the CNS through the olfactory route [158, 159]. A related influenza model, H3N2 influenza A subtype X31, seems to infect sustentacular cells but does not spread to the CNS [160]. This infection causes CD8 T cells to adopt a resident memory phenotype in the OM where they are protected from rechallenge. Unlike CD8 T cells in the lung, OM CD8 T-cell residence is independent of TGF-β, suggesting that different factors are responsible for recruiting or retaining these cells between the lung and olfactory compartments. Importantly, olfactory T cells show limited dissemination to the lungs, protecting against severe disease. IFNs may be important factors for confining infection to the nasal passages, as one study found that Type I and Type III IFNs can prevent murine-adapted influenza A/seal/mass/1-SC35M/1980 (H7N7) and influenza A/duorn/307/1972 (H3N2) from reaching the lungs [161]. Ferret studies similarly indicated differing levels of OM infection between strains, observing no OM infection for A/H3N2/Netherlands/2008 but moderate OM infection in highly pathogenic A/H5N1/Indonesia/2005 and high levels of OM replication in the pandemic H1N1/Netherlands 2009 strain [162]. Hong Kong/**H5N1**/97 also replicated to high levels in ferret nasal turbinates without reaching the brain [163]. Interestingly, a study comparing nasal and lung infection across multiple influenza strains in ferrets indicated that only nasal infection allows for airborne transmission between organisms, while lung infections are not spread [164]. These data indicate that the URT immune response can not only prevent viral spread to the lung but also prevent propagation to other individuals in the population.

Influenza is frequently associated with neurologic symptoms and sequelae [165–167], and in many cases, influenza infection has coincided with meningitis or encephalitis [168–173]. Influenza antigen has been identified in the olfactory nerve of a postmortem sample, lending credibility to the olfactory route of CNS infection [174]. As mentioned above, many influenza strains have olfactory mucosal tropism, and direct olfactory neuroinvasion to the CNS has been reported in several mammalian influenza models. Influenza A/WSN/33 (H1N1) was shown to infect OSNs and spread to the olfactory bulb in immunocompetent mice, and mice lacking an adaptive immune response eventually died [175]. Highly pathogenic avian influenza virus A/Indonesia/5/05 (H5N1) is able to bind to OSN cilia, initiate massive OM infection, and likely uses the olfactory portal to cause CNS pathology in ferrets [162, 176]. Similarly, the pandemic Netherlands 2009 strain of H1N1 was able to infect the olfactory nerves and brains of ferrets. The influenza A/Vietnam/1203/2004 strain resulted in olfactory neuroinvasion and death in ferrets, although in mice, it does not seem to use the olfactory nerve to reach the CNS [163, 177]. In addition, a recent study identified two H3N8 isolates that infected the ferret brain and caused fatality, but the route of neuroinvasion was not analyzed [178]. Much remains to be learned about the viral and host factors that determine the neuroinvasive proclivities of various influenza strains, but the serious potential for olfactory mucosal involvement deserves consideration as new seasonal and pandemic strains emerge.

## Conclusions

The COVID-19 pandemic has led to massive upheaval worldwide. Millions of people have lost their lives, and thousands continue to struggle with the long-term impacts of SARS-CoV-2 infection, including smell loss. When widespread reports surfaced that SARS-CoV-2 infection causes rapid onset smell loss, it signaled the unusual olfactotropic nature of SARS-CoV-2 and foreshadowed the importance of an overlooked and underserved topic of tissue-specific immunity.

Olfactotropic viruses such as SARS-CoV-2, both known and emergent, pose a threat to public health and are an open area of investigation for immunology. Coronaviruses and influenza viruses, the two families that pose the greatest threat for future pandemics, have been shown to variably impact the olfactory mucosa. OM immunity must be considered for microbes that invade the brain through the olfactory nerve but also for pathogens that replicate primarily within the nasal passages. Olfactotropism may be more common than currently believed, as few studies have examined OM infection in great detail for many airborne diseases.

What are the major outstanding questions about olfactory immunology, and what can we do to address these issues? First, we must overturn the dogma that the nasal passages are a single homogenous tissue. The nose contains two distinct epithelial tissues with divergent immune parameters: the respiratory mucosa and the olfactory mucosa. In both animal and human studies, we should increase efforts to delineate and distinguish the two since the immune response fundamentally differs between them. In conjunction with this concept, we should increase olfactory biopsy sampling, particularly in cases of upper respiratory infection. Conventional nasal swabs only capture respiratory tissue, and nasal washes either fail to distinguish respiratory from olfactory tissue or miss the latter entirely. It is likely that many pathogens other than those mentioned above infect the olfactory mucosa, and we have yet to recognize them.

Because the nasal passages are frequently the first to encounter airborne pathogens, the innate immune response in the olfactory mucosa is critical. As a neuronal tissue, the OM innate response may have several key differences when compared to the RM, which is dominated by more classical epithelial cells. Some evidence suggests that the IFN response in this tissue differs from that in the rest of the nasal airway. Immune cells in the OM must balance immune activity with maintaining the neurogenic potential of the olfactory mucosa while avoiding inflammation that may impact the CNS. Resident OM myeloid cells are the first responders to infection, but infiltrating cells likely play important roles in limiting replication.

Few studies have directly addressed adaptive immunity in the OM. One newly identified and critically important consideration for the innate and humoral immune response is the blood-olfactory barrier (BOB). This structure has yet to be directly identified in humans and remains to be further characterized in animal models, but by shaping access of serum proteins to the olfactory tissues, the BOB places limitations on OM immune responses. The presence of the BOB emphasizes the importance of mucosal resident plasma cells that produce locally protective antibodies, as well as the need for local production of large molecular weight molecules (such as complement factors). The dynamics that determine lymphocyte homing, residence, and retention in the OM remain to be fully described and will be important for both B and T-cell-mediated immunity.

Mounting evidence exists to link olfactory inflammation and neurodegenerative disorders, but the molecular and cellular mediators of such a connection remain to be understood. Inflammation of the olfactory mucosa may affect proximal brain structures and contribute to cognitive dysfunction in conditions such as long COVID. Neuroinvasive pathogens that use the olfactory nerve to enter the CNS and the subsequent immune response may breach the barrier to allow progressive infections and inflammation that eventually culminate in neurodegenerative disorders such as Alzheimer’s disease.

Vaccines against both respiratory disease and neurotropic microbes should consider OM protection as critical to their success, both in limiting disease severity and in halting transmission between individuals. To achieve high levels of mucosal antibodies at the olfactory surface, vaccine strategies that induce olfactory-homing plasma cells should be considered. Whether this is best achieved by certain adjuvants, particular antigen formulations, intranasal immunization, or prime-pull vaccination remains to be determined. Similarly, vaccines that induce T cells to reside in the nasal passages are also important for protection, especially against viruses that evolve to escape humoral pressure.

Knowledge of therapeutics that address olfactory infection, both during and after the acute phase of disease, is currently limited. General immunomodulatory approaches to interfere with viral replication, such as intranasal administration of IFN or other cytokines, have been thoroughly investigated only with respect to respiratory regions of the nasal mucosa. Similarly, anti-inflammatory drugs to limit olfactory inflammation may be appropriate for treatment of anosmia or other conditions, such as rhinitis. Steroids, olfactory training, and adoptive stem cell therapies have been investigated for loss of smell [179, 180], but drugs that target immune cells such as neutrophils, macrophages, or T cells might provide an alternative approach given the impact of sustained inflammation. There has been a dramatic increase in patients with long-term smell loss due to SARS-CoV-2 infection, and there is an urgent need for therapeutic interventions to help these individuals [181]. We are just beginning to understand the role of olfactory immunity in infectious disease, but the future promises more exciting advances.
